# Molecular Landscape of Resected Thymomas: Insights from Mutational Profiling

**DOI:** 10.3390/diagnostics16030484

**Published:** 2026-02-05

**Authors:** Luca Frasca, Antonio Sarubbi, Lorenzo Nibid, Ilaria Suriano, Filippo Longo, Giovanna Sabarese, Daniela Righi, Giuseppe Perrone, Pierfilippo Crucitti

**Affiliations:** 1 Department of Thoracic Surgery, Fondazione Policlinico Universitario Campus Bio-Medico, Via Alvaro del Portillo, 200, 00128 Rome, Italy; ilaria.suriano@unicampus.it (I.S.); filippo.longo@policlinicocampus.it (F.L.); p.crucitti@policlinicocampus.it (P.C.); 2 Master’s Degree Program in Medicine and Surgery, Campus Bio-Medico University of Rome, Via Alvaro del Portillo, 21, 00128 Rome, Italy; 3 Research Unit of Anatomical Pathology, Department of Medicine and Surgery, Campus Bio-Medico University of Rome, Via Alvaro del Portillo, 21, 00128 Rome, Italy; lorenzo.nibid@policlinicocampus.it (L.N.); g.perrone@policlinicocampus.it (G.P.); 4 Anatomical Pathology Operative Research Unit, Fondazione Policlinico Universitario Campus Bio-Medico, Via Alvaro del Portillo, 200, 00128 Rome, Italy; g.sabarese@polilcinicocampus.it (G.S.); d.righi@policlinicocampus.it (D.R.)

**Keywords:** thymoma, PD-L1, PIK3CA, thymectomy, recurrence

## Abstract

**Background/Objectives:** Thymomas are the most common tumors of the anterior mediastinum. While early-stage disease often has a favorable prognosis, therapeutic options in advanced stages remain limited. Moreover, the molecular profile of thymomas is still poorly characterized. In the present study, we explored the presence of targetable mutations and programmed death-ligand 1 (PD-L1) expression in a cohort of surgically resected thymomas. Furthermore, we investigated the correlation between PD-L1 expression, histological subtype, and risk of recurrence in patients who underwent curative-intent thymectomy. **Methods:** Mutational profiling was performed using a DNA-based NGS Cancer Panel of 16 genes. PD-L1 expression was evaluated via Tumor Proportion Score (TPS), and thymomas with TPS ≥ 50% were identified as high expressors. The associations with histological subtype and disease-free survival (DFS) were analyzed using logistic regression, Cox proportional hazards models, and Kaplan–Meier survival curves. **Results:** In our study, 2/37 (5.4%) of tested neoplasms (type AB and B2 thymoma) reported as a PIK3CA mutation; no other targetable mutations were observed. Moreover, high PD-L1 expression (≥50%) was reported in (15/37) 40.5% of patients and was significantly associated with aggressive histological subtypes (B2 and B3) (*p* < 0.001). Logistic regression analysis showed that high PD-L1 expression was a significant predictor of aggressive histology (McFadden’s R^2^ = 0.268, *p* < 0.001), with an odds ratio of 15.5 (95% CI: 2.9–83.4; *p* = 0.001). During follow-up, 5/37 (13.5%) of patients experienced disease recurrence; however, no significant difference in DFS was found between high and low PD-L1 expression groups. **Conclusions:** Our data confirm the presence of PIK3CA mutations in thymomas and encourage the exploration the potential role of molecular target therapy in this setting. Moreover, we underlined that high PD-L1 expression level is associated with more aggressive thymoma subtypes and may have a role as a prognostic biomarker. These findings support the need for further studies on the potential role of molecular and predictive pathology in thymic epithelial tumors.

## 1. Introduction

Thymic epithelial tumors (TETs), including thymoma and thymic carcinoma (TC), are the most common mediastinal tumors in adults [1]. Thymomas are the most predominant histotype, frequently associated with a wide range of autoimmune disorders such as myasthenia gravis (MG) [2,3].

Treatment and prognosis are strongly related to the tumor stage. Complete resection is the first choice of treatment for patients with early-stage thymoma; however chemotherapy (CT) alone or associated with radiotherapy (RT) is often used for advanced or metastatic disease [4].

The rarity of thymoma represents a great challenge to the study of the biology of these tumors as well as to the development of novel personalized targeted therapies [5,6]. Mutations and aberrant expression levels of several genes have been identified in thymoma and TC. EGFR is highly expressed in some thymoma and TC samples, while TC more commonly harbors mutations or overexpression of ERBB2, KRAS, TP53, and KIT [7].

Programmed death-ligand 1 (PD-L1) is an immune checkpoint protein that is expressed in tumor cells [8]. Immunohistochemical studies for PD-1 and its ligand performed on tumor tissue can identify patients who are likely to respond to anti-PD-1/PD-L1 drugs. Given the importance of the thymus in the development of the adaptive immune system, there has been interest in examining the potential association between PD-L1 expression and prognosis of patients diagnosed with TETs [9].

Although PD-L1 expression has been reported, its correlation with aggressive histologic subtypes and its prognostic value remains unclear, and long-term follow-up studies are needed [10].

Furthermore, following the positive results achieved in other malignancies, including non-small cell lung cancer (NSCLC), PD-1/PD-L1 immune checkpoint inhibitors could represent a potential complementary or alternative therapeutic option, particularly for more aggressive thymomas [6,8,11]. However, because of the biological characteristics of the thymus, these therapies can also be associated with potentially severe autoimmune toxicity [9].

In this study, we explored the presence of targetable mutations and the association between PD-L1 expression, histologic subtype, and risk of recurrence in patients undergoing extended thymectomy.

## 2. Materials and Methods

Patients referred to our University Hospital (Fondazione Policlinico Universitario Campus Bio-Medico, Rome, Italy) for surgical extended thymectomy were retrospectively analyzed. The study was conducted in accordance with the Declaration of Helsinki and with the approval of the Internal Review Board.

Written informed patient consent was obtained from all patients, and the manuscript adheres to Enhancing the Quality and Transparency of Research (EQUATOR) guidelines.

### 2.1. Study Design

Between September 2018 and March 2025, all patients with suspected thymic lesions underwent preoperative thoracic computed tomography (CT) and 18-fluorodeoxyglucose positron emission tomography/computed tomography ([^18^F]FDG PET/CT) within 30 days prior to surgery.

In some cases, the low-dose (LDCT) scan of participants in the lung cancer screening program were checked and abnormal findings, besides lung nodules, were recorded.

Clinical and surgical data were reviewed for each patient, including the presence or absence of MG, the modified Masaoka–Koga system stage, as well as the 8th TNM stage [4].

Tumor size was measured on CT scans using a cut-off value of 40 mm for classification purposes [12]. PET metabolic activity was evaluated using SUVmax, and because anterior mediastinal solid tumors ≤50 mm with SUVmax ≤ 3–3.4 are frequently low-grade thymomas, this parameter may serve as an oncological criterion for limited resection [13].

In case of doubt, magnetic resonance and fine needle biopsy were performed to determine infiltration of neighboring structures or to obtain a preoperative diagnosis.

All patients underwent extended thymectomy, defined as en bloc resection of the tumor, the entire thymic tissue, the peri-thymic fat, and the upper thymic poles, with the neighboring structures in the case of suspected infiltration. Extended thymectomy including mediastinal adipose tissue was performed in case of MG to optimize neurological outcomes [14].

All procedures were performed under general anesthesia and participants were provided with written informed consent. Surgical approaches consisted of Uniportal Video-Assisted Thoracoscopic Surgery (uVATS), median sternotomy, or thoracotomy. The choice of approach was selected according to preoperative imaging findings and patient-specific characteristics.

Induction therapy was indicated considering preoperative imaging and after multidisciplinary discussion, while adjuvant therapy (AD) was indicated if macroscopically residual of disease (R2) or doubt of microscopically residual (R1) of disease were identified. CT and/or radiotherapy RT were indicated based on tumor characteristics, patient clinical conditions, and previous treatment incidences according to the latest guidelines [4].

All pathological examinations were conducted in the same center’s pathology department. Histological analysis was reviewed on formalin-fixed, paraffin-embedded (FFPE) sections stained with hematoxylin and eosin. Pathological specimens were staged according to the Masaoka–Koga staging system; since 2014, a referral to the TNM staging system has also been reported [15]. Regarding patients operated before the TNM adoption, the pathological report has been reviewed and re-staged according to the TNM [16]. Histology was categorized in accordance with the fifth edition of the WHO classification of Thoracic Tumors (2021 ed.) [17].

Mutations in 16 genes (KRAS, BRAF, EGFR, ALK, FGFR3, KIT, PDGFRA, HRAS, PIK3CA, IDH1, MET, RET, ERBB2, IDH2, NRAS, ROS1) were also analyzed, selected on currently available molecular targeted therapies [18,19,20]. Mutational profiling was performed using the Myriapod NGS Cancer Panel DNA Assay Kit (Diatech Pharmacogenetics, CE-IVD certified; sensitivity and specificity ≥ 99% and LOD ≥ 5%).

According to the most promising data in the literature, PD-L1 expression was retrospectively assessed using the VENTANA PD-L1 (SP263) assay with a Tumor Proportion Score (TPS) cut-off ≥ 50% to identify high expressors [10,21,22,23]. We investigated the correlation of PD-L1 expression with histological thymomas aggressiveness and its prognostic role.

After surgery, all patients were followed up with clinical examination and CT imaging dataevery year for 5 years for stage I/II thymomas according to multidisciplinary tumor board indications in order to determine the presence/absence of recurrence. For R1–R2 resections or stage III thymomas, follow-up consisted of a CT scan every 6 months for 2 years. Patients affected by MG also underwent neurological surveillance (clinical evaluation and lab tests) [2].

### 2.2. Statistical Analysis

All statistical analyses were conducted using Jamovi software, version 2.3.28.0. Descriptive statistics included median and interquartile range (IQR) for continuous variables and frequency counts for categorical variables. Due to the limited sample size, Fisher’s exact test was used to compare PD-L1 expression levels (categorized as <50% vs. ≥50%) with other variables. A *p*-value ≤ 0.050 was considered statistically significant.

To simplify the analysis, certain variables were dichotomized as follows: age below vs. above median, surgical approach in open vs. minimally invasive, BMI: ≤25 (normal weight) vs. >25 [24], comorbidities as absent vs. at least one, stage into early (stage I) vs. advanced (stages II–III), histology into low-grade (A, AB, B1) vs. high-grade (B2, B3) [4].

Disease-free survival (DFS) was analyzed using the Kaplan–Meier method, and the prognostic association between PD-L1 expression and disease control was evaluated using Cox proportional hazards regression. Overall survival (OS) was not assessed, as no patient died from tumor-related causes during the study period.

## 3. Results

A total of 43 patients underwent extended thymectomy in our center. Two cases were diagnosed retrospectively as thymic hyperplasia and four as TC and were, therefore, excluded. Thus, the final study population consisted of a cohort of 37 patients (16 males and 21 females) who met all inclusion criteria. The median age registered was 67 years. Of the patients, 45.95% were non-smokers, while 54.05% had a history of smoking. Hypertension was the most frequently observed comorbidity (59%), while 25% of patients had no comorbidities and 75% had at least one comorbidity. Overall clinical and pathological characteristics are reported in Table 1.

After excluding non-numeric values, the mean tumor diameter measured by CT was 4.9 cm (±2.5), ranging from 1.3 to 10.3 cm. The median diameter was 4.5 cm, and 75% of patients had lesions smaller than 6 cm.

A threshold value of 40 mm was considered, and approximately 64% of tumors had a diameter ≥ 40 mm, while the remaining cases (approximately 36%) had a diameter below this threshold. The maximum standardized uptake value (SUVmax) on PET was dichotomized using a cut-off of 3. Specifically, SUVmax was ≥3 in 48.65% of patients and <3 in 51.35%.

Twenty-three patients (62.16%) received surgery as the sole treatment. The most common surgical approach was uVATS 78.8%, followed by thoracotomy (13.51%) and median sternotomy (8.11%).

AD therapy was indicated if R2 (4/37) or doubt of R1 (10/37) were identified. A total of 14 (37.8%) patients received at least one form of adjuvant treatment among CT, radiotherapy (RT), and chemoradiotherapy (CTRT). Specifically, 10 patients (27.1%) received RT, 3 patients (8.1%) received CT, and only 1 patient (2.7%) received CTRT.

Stage II was the most frequently observed, precisely in 19 patients (51.34%), followed by stage I and stage III in 9 (both 24.33%). WHO histotype frequencies among the study population were as follows: 5 with subtype A (13.5%), 8 with subtype AB (21.6%), 9 with subtype B1 (24.3%), 11 with subtype B2 (29.7%), and 4 with subtype B3 (10.9%).

### 3.1. Mutational Profiling

Only mutations in the PIK3CA gene were detected in two patients (5%): one diagnosed with AB thymoma (PIK3CA: c.1636C>A; p.Gln546Lys; mutation rate 12.39%; Class IV/V—pathogenic/likely pathogenic) and one with B2 thymoma (PIK3CA: c.1258T>C; p.Cys420Arg; mutation rate 11.15%; Class V—pathogenic). No other mutations were found in the other genes analyzed in the remaining cases.

### 3.2. PD-L1 Results

**Figure 1:**
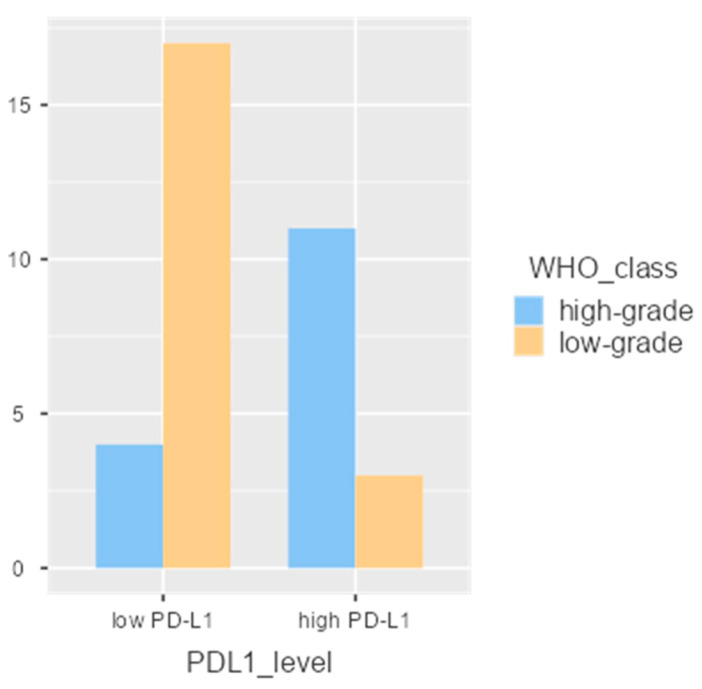
Correlation of low and high PD-L1 expression with WHO histological severity (high/low grades).

High PD-L1 expression was observed in 56.76% of patients (21 out of 37). As shown in Table 2, PD-L1 expression was significantly associated with more aggressive thymoma histological subtypes, particularly those classified as B2 and B3 (*p* < 0.001). When analyzing the distribution of PD-L1 expression across histological subtypes, the following was observed: In type A and AB thymomas, all patients (*n* = 13) had PD-L1 levels < 50%. For B1 and B2 subtypes, PD-L1 ≥ 50% was detected in 44.4% (4/9) and 63.6% (7/11) of the cases, respectively. In type B3 thymomas, all patients (4/4) exhibited PD-L1 expression ≥50%. These findings suggest a trend of increasing PD-L1 expression with higher histological severity, peaking in the aggressive B3 subtype (Figure 1).

Additionally, as shown in Table 3, among patients with MG, there was a higher frequency of PD-L1 ≥ 50%, with a distribution reaching statistical significance on Fisher’s exact test (*p* = 0.066). Although not significant, this observation may be consistent with the underlying immunopathology of MG.

Within the study population, PD-L1 expression did not show statistically significant associations with SUVmax > 3 on PET imaging (*p* = 0.294) nor with the administration of adjuvant therapy following surgery (*p* = 0.093).

**Figure 2:**
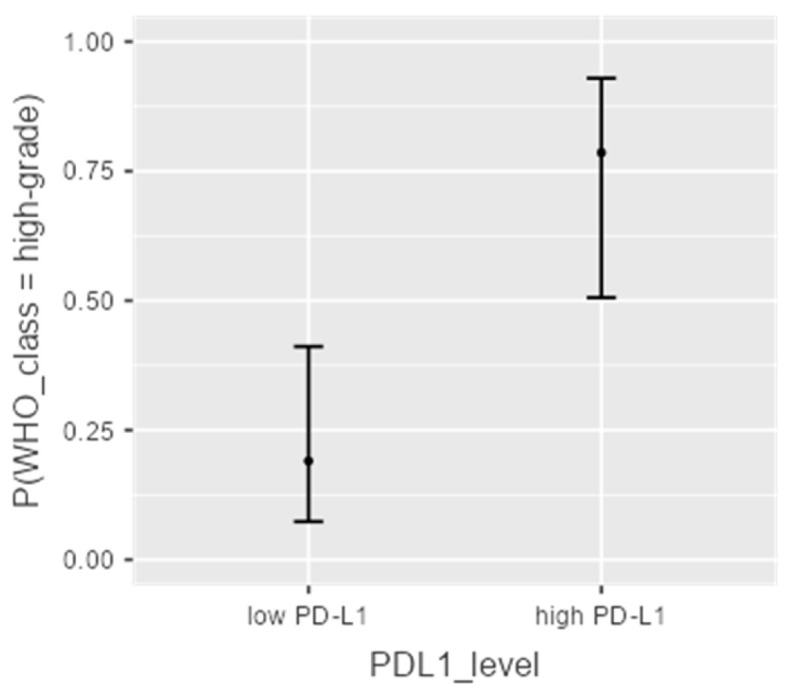
Graphic representation of PD-L1 expression levels related to WHO classes.

A logistic regression model was developed to estimate the predictive value of PD-L1 expression in determining histological subtype aggressiveness. The model was statistically significant (McFadden’s R^2^ = 0.268, *p* < 0.001), with an odds ratio (OR) of 15.5 for PD-L1 expression ≥ 50% (*p* = 0.001; 95% CI: 2.9–83.4), as shown in Figure 2.

**Figure 3:**
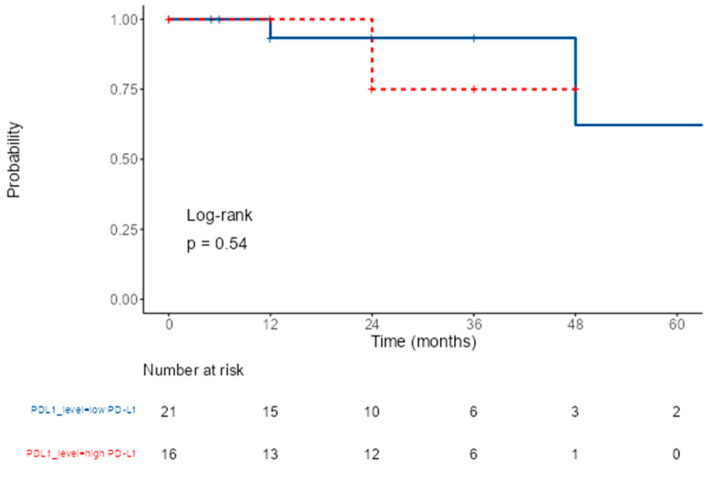
Kaplan–Meier survival analysis for PD-L1 level.

During the follow-up period, 5 out of 37 patients experienced disease recurrence; 3 of whom were in the group with PD-L1 expression ≥ 50%. Kaplan–Meier survival analysis revealed no significant difference in disease-free survival between patients with PD-L1 ≥ 50% and those with <50% (*p* = 0.54) as shown in Figure 3.

This finding was further confirmed by Cox proportional hazards regression analysis, which also showed no statistically significant association (*p* = 0.834).

## 4. Discussion

Over the past decade, technological advancements have enabled the identification of a wide spectrum of molecular aberrations and altered signaling pathways in thymomas. These discoveries have led to the definition of distinct molecular profiles and have paved the way for new targeted therapies.

A recent analysis of comprehensive genomic profiling (CGP) on a cohort of thymomas revealed alterations in genes involved in the cell cycle (CCND3, CDK4/6 and MDM4), DNA damage repair pathways (BRCA1, RAD51C, RAD54L, CHEK2 and MLH1), RTK family signaling dysregulation (FGFR1/4 amplifications), and amplifications in the MYC oncoprotein family (MYC, MYCL and MYCN). There was also evidence of a TP53 oncogenic variant and PI3K/AKT/mTOR pathway activation (ESR1 and PIK3CA) [20]. Similarly, Sakane et al. identified potentially druggable mutations in the KRAS, PIK3CA, and AKT1 genes in a cohort of TC and A/B3 thymomas [18]. Moreover, the first integrated genomic and transcriptomic characterization of a Chinese TET cohort identified recurrent mutations in NF1, ATM, and GTF2I [7].

Interestingly, PIK3CA gene mutations are potentially targetable, and their recurrence in different cohorts of studies suggests that they account for approximately 5% of thymomas. In line with the recent literature, we detected two such mutations in PIK3CA, and to the best of our knowledge, these alterations have not been described previously in an AB or B2 thymoma. Our results support the adoption of a limited molecular panel for thymoma and TC to further explore the role of PIK3CA mutations in these conditions and the potential of molecular targeted therapy for thymoma. At present, there is no evidence regarding the clinical relevance of PIK3CA in thymomas; therefore, data from our series suggest the possibility of investigating, through clinical trials or compassionate-use administration, the potential benefit of anti-PIK3CA therapies in thymomas. Table 4 summarizes reported PIK3CA mutations in thymoma and TC and indicates their associations with targeted therapies approved for these mutations in other malignancies, which have not yet been investigated in thymic tumors [25,26].

Unlike many other malignancies, immunotherapy currently plays a limited role in the treatment of TETs. Despite that, Yokoyama et al. reported high PD-L1 expression in thymomas, especially in higher-grade subtypes (B2/B3) and advanced stages (III/IV), suggesting a better response to anti-PD-1/PD-L1 agents [27]. However, the strong connection between these tumors and the immune system, as well as the potential immune-related adverse events, currently restricts the use of immune checkpoint inhibitors to an off-label context, reserved for patients who have exhausted standard treatment options.

Significative difference in PD-L1 expression level between groups A/AB/B1 and B2/B3 (OR, 0.22; 95% CI, 0.12, 0.41; *p* < 0.001) was found by Hua-Fei Chen et al., with a relatively low heterogeneity (*I^2^* = 55%) underlying the strong correlation of PD-L1 with the most aggressive histologic thymomas [28].

Similarly, Yokoyama et al. demonstrated a statistically significant correlation between high PD-L1 expression and Masaoka stage III/IV disease (*p* = 0.043) as well as B2 or B3 thymoma subtypes (*p* = 0.044) [20]. They also found an association with shorter DFS after surgery (*p* = 0.021), although no impact on OS was observed (*p* = 0.957) [29].

More recently, Chubachi and colleagues evaluated PD-L1 expression in thymomas and TC using SP142 and SP263 antibodies, demonstrating antibody-dependent expression rates and higher PD-L1 positivity in type B thymomas [30].

Our findings support this trend, showing increased PD-L1 expression in B2/B3 thymomas but not in tumors that required adjuvant treatment after surgery, suggesting an active role of immune evasion mechanisms in the more aggressive subtypes. In fact, nearly all patients who experienced recurrence in our cohort showed PD-L1 expression levels above 50%, and multivariate Cox regression analysis confirmed PD-L1 ≥50% as a predictor of worse DFS, along with histology and disease stage. These findings confirm the results of a previously published multicentric study by Frasca et al. on a broader cohort of patients [22].

The correlation between high PD-L1 expressions could reveal important perspectives for immunotherapy. Several studies were conducted on the response of the tumor to pembrolizumab treatment, which highlighted significative immune-related adverse events, suggesting the need for further investigation into toxicity management. Despite promising results, the off-label status of immunotherapy in thymomas complicates its clinical use and requires careful monitoring of side effects [31].

Currently, pembrolizumab is an off-label option for patients who have already received at least one line of platinum-based CT [32].

This study has several limitations. Firstly, it is a retrospective analysis. In addition, it is a pilot study with a relatively small sample size that should be considered exploratory and hypothesis-generating. Nevertheless, our results align with previous studies in the literature and underscore the potential role of PD-L1 as a prognostic biomarker.

## 5. Conclusions

In conclusion, thymomas are rare and often enigmatic tumors that may present at advanced stages or in younger patients. Our study explored genetic mutations and PD-L1 overexpression, highlighting their potential roles in guiding targeted therapies and predicting tumor aggressiveness. More specifically, the identification of PIK3CA mutations could, in the future, enable new therapeutic options for patients with thymoma, while the presence of PD-L1 overexpression could aid in prognostic stratification, together with the accurate definition of histologic subtype and disease stage. Despite the small cohort, the identification of actionable mutations underscores the clinical relevance of molecular profiling. In this era of precision oncology, expanding research on immunotherapy in thymic tumors is crucial. Larger multicentric studies are needed to validate these findings and develop new treatment options.

## Figures and Tables

**Table 1:** Demographic and pathological characteristics of the study population.

Variable	
Age (years), median (IQR ^1^)	67 (23–82)
Gender, *n* (%)	
Male	16 (43.24%)
Female	21 (56.76%)
BMI ^2^ group, *n* (%)	
Normal (18.5–24.99 kg/m^2^)	16 (43.24%)
Overweight (25–29.99kg/m^2^)	12 (32.43%)
Obese (30–39.99 kg/m^2^)	9 (24.33%)
Smoke history, *n* (%)	
No	17 (45.95%)
Yes	20 (54.05%)
CT ^3^ diameter (mm), median (IQR)	49 (13.0–103.0)
CT diameter cut-off, *n* (%)	
≤40 mm	24 (64.86%)
≥40 mm	13 (35.14%)
SUVmax ^4^, *n* (%)	
≤3	19 (51.35%)
>3	18 (48.65%)
Treatment, *n* (%)	
Surgery alone	23 (62.16%)
Adjuvant therapy	14 (37.84%)
Radiotherapy	10 (27.1%)
Chemotherapy	3 (8.1%)
Chemoradiotherapy	1 (2.7%)
Surgical approach, *n* (%)	
VATS ^5^	29 (78.38%)
Sternotomy	3 (8.11%)
Thoracotomy	5 (13.51%)
WHO ^6^ Histology, *n* (%)	
A	5 (13.52%)
AB	8 (21.62%)
B1	9 (24.32%)
B2	11 (29.72%)
B3	4 (10.82%)
Masaoka–Koga Stage, *n* (%)	
Stage I	9 (24.33%)
Stage II	19 (51.34%)
Stage III	9 (24.33%)

^1^ IQR: interquartile range, ^2^ BMI: body mass index, ^3^ CT: computed tomography, ^4^ SUV: standard uptake value, ^5^ VATS: video-assisted thoracic surgery, ^6^ WHO: World Health Organization.

**Table 2:** WHO type frequencies stratified for low and high PD-L1 level.

WHO ^1^ Type	PD-L1 ^2^ Level	Frequencies	% of Total	% Cumulative
A	L ^3^ PD-L1	5	13.5%	13.5%
	H ^4^ PD-L1	0	0.0%	13.5%
AB	L PD-L1	8	21.6%	35.1%
	H PD-L1	0	0.0%	35.1%
B1	L PD-L1	5	13.5%	48.6%
	H PD-L1	4	10.8%	59.4%
B2	L PD-L1	4	10.8%	70.2%
	H PD-L1	7	19.0%	89.2%
B3	L PD-L1	0	0.0%	89.2%
	H PD-L1	4	10.8%	100.0%

^1^ WHO: World Health Organization; ^2^ PD-L1: programmed death-ligand 1; ^3^ L: low; ^4^ H: high.

**Table 3:** Association between PD-L1 expression and myasthenia gravis (MG).

PD-L1 Level ^1^	M G		
	No	Yes	Total
Low PD-L1 (<50%)	20 (17.6)	1 (3.4)	21
High PD-L1 (≥50%)	11 (13.4)	5 (2.6)	16
Total	31	6	37

PD-L1 ^1^: programmed death-ligand 1. Expected counts are shown in parentheses.

**Table 4:** PIK3CA mutations reported in thymoma and thymic carcinoma.

PIK3CA Mutation	Protein Domain	Potentially Available Targeted Drugs
p.E542K	Helical	Alpelisib, Taselisib, Buparlisib, Gedatolisib, Everolimus, Temsirolimus
p.E545K	Helical	Alpelisib, Taselisib, Buparlisib, Gedatolisib, Everolimus
p.Q546K	Helical	Alpelisib, Taselisib, Buparlisib
p.C420R	C2	Alpelisib, Buparlisib
p.H1047R	Kinase	Alpelisib, Inavolisib, RLY-2608, CYH33
p.H1047L	Kinase	Alpelisib, Inavolisib, RLY-2608, CYH33

## Data Availability

The data presented in this study are available on request from the corresponding author due to privacy.
